# Flumazenil reversal of remimazolam-induced sedation: a narrative review of safety, pharmacokinetics, and clinical considerations

**DOI:** 10.3389/fmed.2026.1793528

**Published:** 2026-04-20

**Authors:** Yukai Zhou, Wenzhi Wu, Yi Zhang, Yanhua Peng, Wencai Jiang, XianJie Zhang, Feng Ju, An Xie

**Affiliations:** 1 Department of Anesthesiology, Number 1 Orthopedics Hospital of Chengdu, Chengdu, China; 2 Department of Anesthesiology, Wanzhou District Maternal and Child Health Hospital of Chongqing, Chongqing, China; 3 Anesthesiology and Critical Care Medicine Key Laboratory, Southwest Medical University, Luzhou, China; 4 Department of Anesthesiology, Deyang People’s Hospital, Deyang, China

**Keywords:** benzodiazepine reversal, drug safety, flumazenil, GABA-A receptor, pharmacodynamics, pharmacokinetics, remimazolam, re-sedation

## Abstract

**Introduction:**

Remimazolam, an ultra-short-acting benzodiazepine metabolized by carboxylesterase-1 (CES1), permits specific antagonism by flumazenil, enabling active reversal unavailable with propofol-based sedation. However, the safety profile of this reversal strategy-including re-sedation risk, seizure concerns, and special population considerations-remains incompletely characterized.

**Methods:**

This narrative review synthesizes evidence from randomized controlled trials, meta-analyses, pharmacokinetic-pharmacodynamic modeling studies, and pharmacogenomic research identified through comprehensive searches of PubMed, Embase, the Cochrane Library, and Google Scholar through February 2026 to evaluate the clinical utility and safety considerations of flumazenil reversal in remimazolam-based anesthesia.

**Results:**

Recent meta-analyses demonstrate that remimazolam-flumazenil accelerates emergence by approximately 4 min versus propofol with significant reductions in respiratory depression (RR 0.41; 95% CI 0.30–0.56) and hypotension (RR 0.25; 95% CI 0.12–0.52), though substantial heterogeneity (*I*^2^ = 96%) limits pooled estimate precision. Re-sedation occurs in 2–22% of cases depending on procedural duration and outcome definitions, with this variability primarily reflecting heterogeneous procedural settings and inconsistent outcome definitions rather than pharmacogenomic factors. The pharmacogenomics of CES1, particularly the G143E loss-of-function polymorphism, represents an emerging area that may influence remimazolam metabolism and reversal kinetics. Reconciliation of surgical database evidence with elevated pharmacovigilance signals from FAERS analysis suggests confounding by indication in emergency settings; however, the intrinsic neurophysiological risks of rapid GABA-A receptor de-occupation warrant continued vigilance. The Dextran 40 excipient in remimazolam besylate formulations is contraindicated in patients with severe dextran hypersensitivity, and clinicians should consider non-benzodiazepine etiologies when hemodynamic deterioration does not respond to flumazenil. In neonates, immature CES1 activity combined with reduced renal clearance creates theoretical risk of metabolite accumulation, contraindicating use outside research settings.

**Discussion:**

This review identifies critical evidence gaps—including the need for standardized re-sedation definitions, prospective validation of pharmacokinetic-pharmacodynamic models, and pediatric pharmacokinetic data—and provides evidence-based considerations for clinical practice while emphasizing the need for systematic review methodology and expert consensus to develop formal clinical guidelines.

## Introduction

The capacity to terminate sedation predictably and on demand represents a longstanding objective in anesthetic pharmacology. Propofol, despite favorable context-sensitive pharmacokinetics, lacks a specific antagonist, rendering emergence timing dependent on redistribution and metabolism rather than active intervention (1, 2). This limitation poses particular challenges in outpatient procedures, high-risk patient populations, and situations requiring rapid neurological assessment.

Remimazolam (CNS 7056) was engineered through “soft drug” design: a methyl propionate ester moiety susceptible to hydrolysis by carboxylesterase-1 (CES1) yields CNS 7054, an inactive metabolite with markedly reduced receptor affinity (3, 4). This esterase-dependent pathway operates independently of hepatic cytochrome P450 enzymes, offering generally predictable pharmacokinetics, with notable exceptions in patients carrying CES1 loss-of-function polymorphisms or receiving CES1-inhibiting medications (5, 6). The intact benzodiazepine pharmacophore permits competitive antagonism by flumazenil, providing a reversal capability absent in propofol-based sedation protocols.

The clinical adoption of remimazolam has expanded rapidly since regulatory approvals in Japan (January 2020), the United States and China (July 2020), Europe (March 2021), and South Korea (August 2021) (7, 8). Multiple systematic reviews and meta-analyses have subsequently evaluated remimazolam’s efficacy and safety profile, particularly in gastrointestinal endoscopy settings where procedural sedation requirements are well-defined (9–12). These analyses consistently demonstrate favorable cardiorespiratory safety profiles compared with propofol, though the optimal integration of flumazenil reversal into clinical practice remains incompletely characterized.

However, the safety profile of this reversal strategy remains incompletely characterized. Re-sedation rates ranging from 0 to 22% across studies raise questions about optimal dosing and monitoring protocols (13–15). Recent pharmacogenomic research has revealed that CES1 genetic polymorphisms significantly impact remimazolam metabolism, introducing additional complexity in predicting individual patient responses (16). This narrative review addresses: (1) What pharmacokinetic and pharmacodynamic principles govern re-sedation risk? (2) How do CES1 pharmacogenomics and drug-drug interactions influence remimazolam metabolism and reversal kinetics? (3) Do historical concerns regarding flumazenil-associated seizures apply to surgical populations? (4) What differential diagnostic considerations are relevant when patient deterioration does not respond to flumazenil?

Literature search strategy: This narrative review is based on a comprehensive literature search of PubMed, Embase, the Cochrane Library, and Google Scholar conducted initially through August 2025, using the search terms “remimazolam,” “flumazenil,” “benzodiazepine reversal,” and “re-sedation” in various combinations. The search was updated through February 2026 during manuscript revision, incorporating newly published randomized controlled trials, pharmacovigilance analyses, and case reports that substantially expanded the evidence base for flumazenil reversal. Randomized controlled trials, systematic reviews, meta-analyses, pharmacokinetic modeling studies, pharmacogenomic research, pharmacovigilance analyses, and regulatory documents were prioritized. Reference lists of included articles were manually searched for additional relevant publications. As a narrative review, this work is subject to selection bias inherent in non-systematic evidence synthesis and does not employ formal quality assessment per PRISMA methodology. One systematic review by Wu et al. (11) has specifically addressed remimazolam-flumazenil reversal versus propofol (11); the present narrative review complements this work by providing broader contextual analysis of pharmacological mechanisms, safety considerations, and special populations. Readers should interpret conclusions with this methodological limitation in mind.

## Pharmacological foundations

### Remimazolam pharmacology and receptor mechanisms

Remimazolam functions as a positive allosteric modulator of GABAA receptors, the principal inhibitory neurotransmitter system in the mammalian central nervous system (17, 18). Upon binding to the benzodiazepine allosteric site located at the interface between α and γ subunits, remimazolam enhances the receptor’s response to endogenous GABA, potentiating chloride conductance and inducing cell membrane hyperpolarization (19, 20). This mechanism results in inhibition of neuronal activity, producing the characteristic benzodiazepine effects of sedation, anxiolysis, anterograde amnesia, and anticonvulsant activity.

The GABAA receptor exhibits remarkable heterogeneity, with different subunit combinations conferring distinct pharmacological properties and anatomical distributions. Remimazolam demonstrates affinity for α 1-, α 2-, α 3-, and α5-containing receptor subtypes without demonstrating clear selectivity among them (3, 21). The α1 subtype predominantly mediates sedative and amnestic effects, while α2 and α3 subtypes contribute to anxiolytic and muscle relaxant properties (22). This non-selective binding profile is consistent with other classical benzodiazepines and distinguishes remimazolam from newer subtype-selective compounds under investigation.

Pharmacodynamic studies utilizing electroencephalographic parameters have characterized remimazolam’s concentration-effect relationships. The effect-site equilibration rate constant (ke0) has been estimated at approximately 0.3–0.5 min^–1^, corresponding to a time-to-peak effect of 2–3 min following intravenous administration (23, 24). This rapid onset, comparable to propofol, enables precise titration during procedural sedation. The pharmacodynamic half-life is approximately 7–8 min, reflecting the drug’s rapid redistribution from the effect site (25).

### CES1-mediated metabolism and pharmacokinetics

The distinguishing pharmacological feature of remimazolam is its ester linkage, which undergoes rapid hydrolysis by carboxylesterase-1 (CES1) to yield the carboxylic acid metabolite CNS 7054 (3, 26). CES1 is the most abundantly expressed drug-metabolizing enzyme in human liver, comprising approximately 1% of the total liver proteome and accounting for 80–95% of hepatic hydrolytic activity (27, 28). This metabolic pathway operates independently of cytochrome P450 enzymes, minimizing the potential for pharmacokinetic drug-drug interactions with the numerous medications metabolized by the CYP system.

Population pharmacokinetic analyses have characterized remimazolam disposition using three-compartment models. Published parameters include: systemic clearance 68 ± 12 L/h (approximately 1.0–1.1 L/min), central volume of distribution 35.4 L, and terminal elimination half-life 37–53 min (14, 23, 29). The context-sensitive half-time is approximately 7–8 min and remains relatively constant across infusion durations of up to 4 h (14), distinguishing remimazolam from other benzodiazepines such as midazolam, which demonstrates substantial accumulation with prolonged administration (30).

Importantly, remimazolam is predominantly hydrolyzed in the liver; recent *in vitro* studies demonstrated that remimazolam was not hydrolyzed by human serum or intestinal S9 fractions, confirming that hepatic CES1 is the predominant enzymatic pathway for its deactivation, with minor contributions from hydroxylation and glucuronidation of the metabolite CNS 7054 (6, 16). This tissue-specific metabolism has implications for patients with hepatic dysfunction, where clearance may be substantially reduced. Studies in patients with Child-Pugh C cirrhosis demonstrated approximately 75% reduction in remimazolam clearance, necessitating dose adjustment and extended monitoring (31).

**FIGURE 1:**
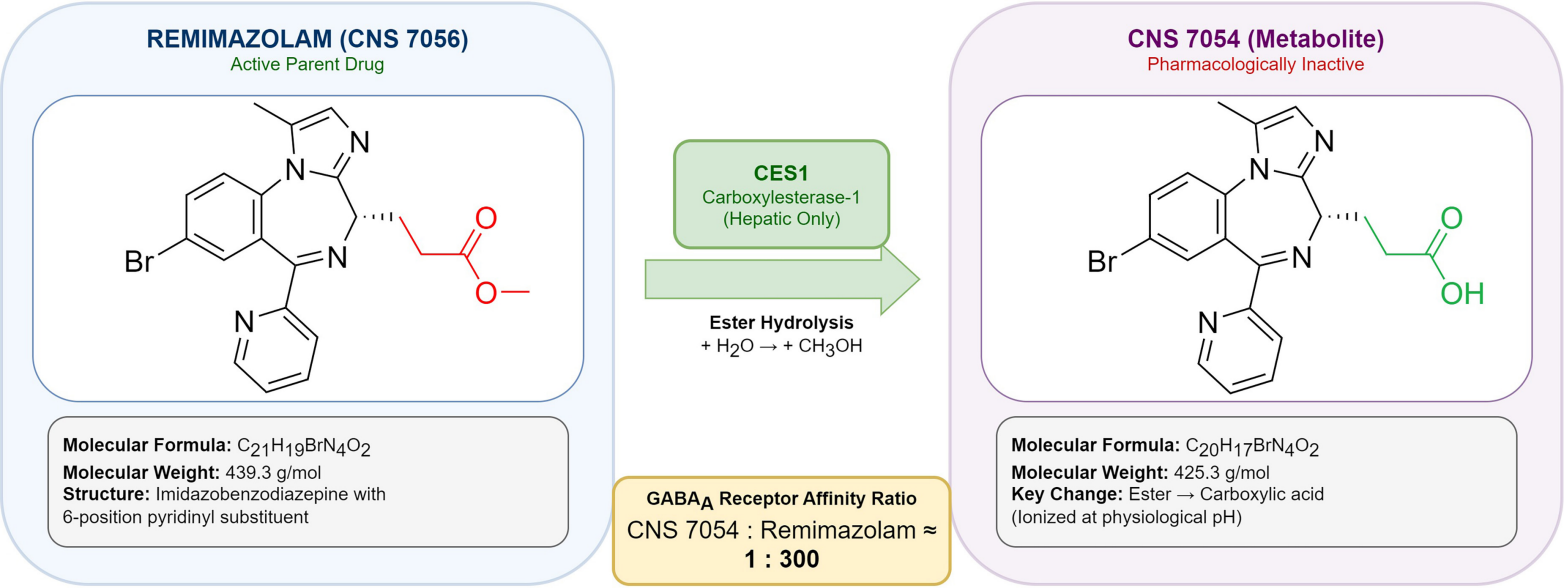
CES1-mediated metabolic pathway of remimazolam. Schematic representation of remimazolam metabolism. Remimazolam (CNS 7056; C21H19BrN4O2, MW 439.3), an imidazobenzodiazepine with a characteristic pyridine ring, undergoes rapid hydrolysis by carboxylesterase-1 (CES1) at the methyl propionate ester bond. This yields the carboxylic acid metabolite CNS 7054 (C20H17BrN4O2, MW 425.3), which has approximately 300-fold reduced affinity for the GABAA receptor benzodiazepine binding site. The metabolite is eliminated renally; this pathway operates independently of hepatic cytochrome P450 enzymes (3).

The metabolite CNS 7054 possesses approximately 300-fold lower affinity for the GABAA receptor benzodiazepine binding site compared with the parent compound (3). This dramatic reduction in receptor affinity effectively renders CNS 7054 pharmacologically inactive at typical plasma concentrations achieved during clinical use. The metabolite is eliminated primarily via renal excretion, with approximately 50–60% of the administered dose recovered in urine as CNS 7054 (32). Renal impairment does not significantly affect parent drug pharmacokinetics, though metabolite accumulation may occur with repeated dosing in patients with severely reduced glomerular filtration rate (Figure 1).

### CES1 Pharmacogenomics and interindividual variability

The CES1 gene exhibits significant genetic polymorphism, with several variants demonstrating functional consequences for drug metabolism (33, 34). Recent research has specifically characterized the impact of CES1 genetic variation on remimazolam hydrolysis, providing important insights into sources of interindividual pharmacokinetic variability (16).

The G143E polymorphism (rs71647871) represents the most clinically significant CES1 variant, functioning as a loss-of-function mutation that substantially impairs enzyme activity (35, 36). Individuals heterozygous for G143E demonstrate approximately 50% reduction in CES1 activity, while homozygosity (extremely rare, < 0.2% prevalence) would theoretically result in near-complete loss of CES1-mediated hydrolysis. Wang et al. (16) verified that liver samples from G143E carriers exhibited significantly reduced remimazolam hydrolysis rates compared with non-carriers, confirming the clinical relevance of this polymorphism for remimazolam metabolism (16).

Beyond genetic polymorphisms, CES1 activity can be modulated by drug-drug interactions with other CES1 substrates or inhibitors. Wang et al. identified that clopidogrel significantly inhibited remimazolam hydrolysis at clinically relevant concentrations, suggesting potential for pharmacokinetic interactions in patients receiving dual therapy (16). Other medications including simvastatin, enalapril, and sacubitril were also evaluated, though their inhibitory effects were less pronounced. The combination of CES1 genetic variants with interacting drugs may produce compounded effects on remimazolam metabolism, particularly in populations receiving multiple cardiovascular medications.

Clinical implications for reversal practice: While CES1 pharmacogenomic testing is not currently recommended for routine clinical practice, these findings have direct relevance to flumazenil reversal. Reduced CES1 activity (whether genetic or drug-induced) would slow remimazolam clearance, potentially widening the vulnerability window for re-sedation following flumazenil administration. Patients who demonstrate unexpectedly prolonged sedation or require higher than anticipated flumazenil doses for reversal may represent individuals with reduced CES1 activity. Importantly, incremental flumazenil titration provides adequate safety margins without genetic testing, but future research may identify clinical scenarios where pharmacogenomic guidance enhances reversal outcome prediction.

### Flumazenil pharmacology

Flumazenil (Ro 15-1788) is a competitive antagonist at the benzodiazepine binding site of GABAA receptors, displacing agonists through higher binding affinity without producing intrinsic receptor activation (37, 38). This mechanism enables rapid reversal of benzodiazepine-induced sedation while preserving normal GABAergic neurotransmission. Flumazenil demonstrates high selectivity for the central benzodiazepine receptor with minimal activity at peripheral benzodiazepine binding sites.

Pharmacokinetic parameters for flumazenil include: rapid onset within 1–2 min following intravenous administration, peak effect at 6–10 min, and elimination half-life of 40–80 min (39, 40). The relatively short duration of action compared with many benzodiazepine agonists underlies the risk of re-sedation, as antagonist plasma concentrations may decline while agonist concentrations remain pharmacologically significant.

The effect-site equilibration rate constant (ke0) for flumazenil is approximately 0.1–0.2 min^–1^, which differs from remimazolam’s ke0 of 0.3–0.5 min^–1^ (23, 41). This difference in blood-brain equilibration rates contributes to complex reversal dynamics, as the temporal profiles of agonist and antagonist effect-site concentrations do not perfectly align. Understanding these kinetic differences is essential for optimizing reversal protocols and predicting re-sedation risk.

## Pharmacokinetic and pharmacodynamic considerations in reversal

### Pharmacokinetic mismatch theory and re-sedation mechanisms

Masui’s pharmacokinetic simulations revealed a counterintuitive relationship between flumazenil dose and re-sedation probability, providing a theoretical framework for understanding reversal dynamics (42). The mechanistic basis involves several interrelated phenomena:

First, high-dose flumazenil produces rapid, near-complete displacement of remimazolam from GABAA receptors, resulting in abrupt arousal. Second, following cessation of remimazolam infusion, drug sequestered in peripheral compartments (adipose tissue, muscle) continues to redistribute back into the central circulation. Third, flumazenil’s rapid redistribution causes antagonist effect-site concentrations to decline faster than ongoing remimazolam elimination can reduce agonist levels. This creates a temporal “vulnerability window” during which receptor occupancy by remimazolam may increase despite overall declining plasma concentrations.

**FIGURE 2:**
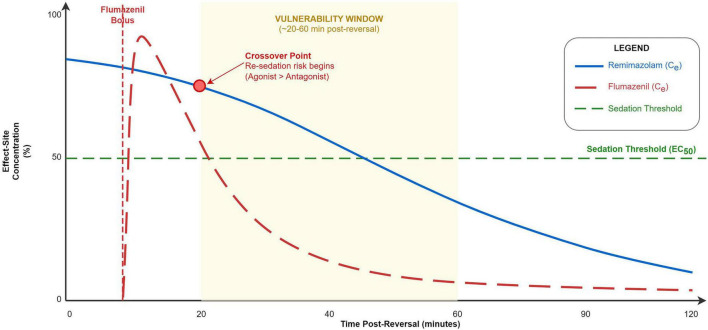
Conceptual PK-PD model of re-sedation risk. Illustration of factors contributing to re-sedation, integrating pharmacokinetic (redistribution, elimination) and pharmacodynamic (receptor occupancy, effect-site equilibration hysteresis) components. The “vulnerability window” is a theoretical construct from simulation (42) without prospective clinical validation.

The simulations predict that this vulnerability window occurs approximately 20–60 min following flumazenil administration, with risk magnitude proportional to cumulative remimazolam dose, infusion duration, and flumazenil bolus size. Paradoxically, larger flumazenil doses may increase re-sedation risk by producing more complete initial reversal followed by more rapid antagonist washout (42, 43) (Figure 2).

Critical limitations: These simulations assume linear elimination kinetics and do not account for potential non-linearity or real-world variability in CES1 activity. The models have not been prospectively validated against clinical respiratory events or other objective re-sedation endpoints. The predicted “mismatch window” remains theoretical and requires clinical confirmation.

### Pharmacodynamic factors contributing to re-sedation

Re-sedation cannot be fully explained by pharmacokinetic factors alone; pharmacodynamic considerations contribute substantially to reversal complexity:

Effect-site equilibration hysteresis: Remimazolam and flumazenil exhibit different ke0 values, creating hysteresis between plasma concentrations and clinical effect. The “mismatch” between agonist and antagonist kinetics is therefore both kinetic (blood concentration) and dynamic (receptor-site occupancy). This hysteresis means that clinical effect may not directly correspond to measured plasma drug levels (24, 44).

GABAA receptor binding dynamics: Competitive antagonism involves complex receptor kinetics. Association and dissociation rate constants for flumazenil and remimazolam at the benzodiazepine site may differ, affecting antagonism durability. The receptor microenvironment, including lipid membrane composition and receptor subunit composition, may influence drug-receptor interactions in ways not captured by simple equilibrium binding models (19).

Acute tolerance and receptor adaptation: Whether repeated flumazenil dosing induces tachyphylaxis through receptor desensitization or internalization remains unknown. GABAA receptors undergo dynamic regulation in response to sustained agonist or antagonist exposure, though the timescale of these adaptations during acute clinical use has not been characterized (45).

Individual variability in receptor sensitivity: Patients demonstrate substantial interindividual variability in benzodiazepine sensitivity, influenced by age, comorbidities, concurrent medications, and potentially genetic factors affecting GABAA receptor expression or function (46, 47).

### Opioid interaction and the unmasking phenomenon

Remimazolam and opioids exhibit supra-additive pharmacodynamic interaction for respiratory depression, a clinically important consideration given that most procedural sedation protocols combine benzodiazepines with opioid analgesics (48, 49). When flumazenil reverses the benzodiazepine component of combined sedation, it removes this synergistic interaction, resulting in the unmasking of residual opioid effects.

Consider, for example, a patient who receives remimazolam combined with fentanyl for endoscopic sedation and, upon flumazenil administration, initially awakens but subsequently develops respiratory depression or appears persistently sedated. In this clinical scenario, the patient is not truly “re-sedated” by benzodiazepine recurrence but rather experiences the unmasking of underlying opioid-mediated respiratory depression that was previously obscured by the synergistic drug combination. Vellinga et al. (50) provided the definitive pharmacokinetic-pharmacodynamic characterization of this interaction, demonstrating through a three-period crossover study in 24 healthy volunteers that remifentanil produces supra-additive synergy with remimazolam on all sedation endpoints, including BIS suppression, loss of responsiveness, and respiratory depression (50). This mechanism is clinically and mechanistically distinct from true pharmacokinetic re-sedation caused by agonist-antagonist mismatch. The clinical implication is direct: if respiratory depression persists after adequate flumazenil reversal, clinicians should consider naloxone administration for opioid antagonism rather than additional flumazenil doses. Attributing post-reversal sedation automatically to benzodiazepine re-emergence may lead to inappropriate management and delayed recognition of opioid toxicity (51).

The pharmacokinetic-pharmacodynamic principles outlined above provide the theoretical framework for interpreting clinical outcomes. The following section synthesizes the growing body of meta-analytic and trial-level evidence, organized by the consistency of findings across studies rather than as a sequential enumeration of individual analyses.

## Clinical evidence

### Meta-analytic findings

The remimazolam literature has accumulated a remarkable density of meta-analytic evidence, with over twenty systematic reviews published since 2022, collectively encompassing more than 14,000 participants across diverse procedural settings (9–12, 52, 53). Synthesizing this body of evidence reveals a consistent pattern: cardiorespiratory safety outcomes converge strongly across analyses, whereas time-based outcomes (emergence time, recovery duration) exhibit extreme heterogeneity that is now well explained by methodological and clinical factors.

Regarding cardiorespiratory safety, the evidence is remarkably consistent. Across all published meta-analyses, remimazolam demonstrates significantly reduced respiratory depression compared with propofol, with risk ratios ranging from 0.35 to 0.47 (9–12, 53). GRADE-assessed evidence quality is rated high for respiratory depression (RR 0.41; 95% CI 0.30–0.56) and hypotension requiring treatment (RR 0.25; 95% CI 0.12–0.52) in the largest endoscopy-focused analysis by Barbosa et al. (9), encompassing 15 trials with 4,516 participants (9). The most comprehensive meta-analysis to date by Pingel et al. (53), analyzing 63 randomized controlled trials with 13,953 participants and applying trial sequential analysis, confirmed these safety advantages with adjusted confidence intervals: respiratory complications RR 0.47 (97.5% CI 0.36–0.61) and cardiovascular complications RR 0.46 (97.5% CI 0.37–0.56) versus active comparators (53). Hemodynamic benefits are equally robust, with An et al. (12) reporting consistent reductions in hypotension (RR 0.49; 95% CI 0.40–0.61) and bradycardia (RR 0.53; 95% CI 0.37–0.76) across 26 trials involving 3,641 patients (12).

In contrast, time-based outcomes exhibit extreme heterogeneity (*I*^2^ = 83–98%) across analyses, and three specific factors now explain this variance. First, whether flumazenil was administered is a critical modifier: Song et al. (54), analyzing 19 trials with 3,031 patients, demonstrated that without flumazenil, remimazolam produced prolonged extubation and PACU stay compared with propofol, whereas with flumazenil reversal, remimazolam achieved significantly shorter emergence (54). Second, Wu et al. (11) formally identified through meta-regression that definitional heterogeneity—specifically, inconsistent criteria for measuring emergence time—was the principal source of between-study variance in their analysis of nine trials (*I*^2^ = 96%) (11). Third, mixing procedural sedation populations with general anesthesia cohorts introduces conceptual heterogeneity, as recovery dynamics differ fundamentally between these contexts (55). Re-sedation rates similarly resist meaningful pooling, with Wu et al. (11) reporting 9.03% versus 2.38% for remimazolam-flumazenil versus propofol (RR 4.15; 95% CI 1.31–13.13) (11), though the clinical significance of this finding is moderated by the definitional challenges discussed below.

Importantly, these safety advantages are preserved in higher-risk subpopulations. Multiple meta-analyses focusing specifically on elderly patients (≥ 60 years) have confirmed consistent reductions in respiratory and hemodynamic adverse events (52, 56), and recent network meta-analyses including over 14,000 patients across 76 trials have identified remimazolam-based regimens among the safest combinations for endoscopic sedation (57). However, as Pingel et al. (55) emphasized, GRADE certainty remains very low to low for most outcomes, and all included trials were judged at high risk of bias, underscoring the need for methodological rigor in future studies (53).

In summary, the meta-analytic evidence establishes remimazolam’s cardiorespiratory safety advantage with high consistency, while revealing that efficacy comparisons—particularly emergence time—are critically dependent on whether flumazenil reversal is employed, how outcomes are defined, and the procedural context studied. This synthesis underscores that the clinical value of remimazolam cannot be fully evaluated independently of its reversal agent, a theme that recurs throughout this review.

### Re-sedation heterogeneity and definitional challenges

Re-sedation rates reported across studies range from 0 to 22%. This variability is primarily attributable to inconsistent outcome definitions, variable monitoring durations, and heterogeneous procedural settings (including marked differences in infusion duration and total drug exposure) rather than pharmacogenomic or drug-drug interaction factors, for which clinical evidence of impact on re-sedation remains lacking. Meta-regression by Wu et al. (11) formally identified definitional heterogeneity as the principal source of between-study variance (11). This variability precludes meaningful pooling of re-sedation data and limits the precision of risk estimates.

Oh et al. (15) reported the highest re-sedation rate (22%) in patients undergoing radiofrequency catheter ablation for atrial fibrillation, procedures characterized by prolonged total intravenous anesthesia durations averaging 2–3 h (15). This elevated rate likely reflects the pharmacokinetic principles outlined previously: extended infusions result in greater peripheral compartment accumulation, increasing the magnitude and duration of redistribution following infusion cessation. Most recently, He et al. (58) presented a comprehensive case report and literature review documenting re-sedation occurring between 13 min and 6 h 35 min post-flumazenil, with the longest episode lasting 12 h; pharmacokinetic simulation demonstrated that neither single large-dose nor divided small-dose flumazenil strategies reliably prevented re-sedation (58). De Jong et al. (23) provided the most comprehensive pharmacokinetic-pharmacodynamic review to date, reporting re-sedation in 3.1% of patients across reviewed studies, and identifying the critical mechanistic insight that larger flumazenil bolus doses combined with lower remimazolam clearance most strongly predict re-sedation risk (23).

In contrast, studies examining short procedural sedation (< 30 min) report re-sedation rates at or below 5%, consistent with limited peripheral drug accumulation (59, 60). Schüttler et al. (14) reported a 3.1% re-sedation rate in healthy volunteers using objective electroencephalographic and MOAA/S-based definitions (14).

The absence of standardized re-sedation definitions represents a critical methodological limitation across the literature. Proposed definitions have included: decrease in modified Observer’s Assessment of Alertness/Sedation (MOAA/S) score of ≥ 2 points from post-reversal baseline, Richmond Agitation-Sedation Scale (RASS) decrease ≥ 2 levels, requirement for additional flumazenil dosing, respiratory intervention requirement, or prolonged PACU stay attributable to sedation. Consensus regarding standardized outcome definitions, potentially achieved through Delphi methodology, would substantially enhance comparability across future studies (61).

### Emergence time comparisons with propofol

Multiple randomized controlled trials have directly compared emergence profiles between remimazolam with flumazenil reversal and propofol-based anesthesia. Lee et al. (62) compared recovery profiles following open thyroidectomy, demonstrating significantly shorter time to eye opening in the remimazolam-flumazenil group (3.2 ± 1.8 vs. 5.8 ± 2.4 min, *p* < 0.01) with reduced variability in recovery times (62). The consistency of emergence timing with remimazolam-flumazenil may facilitate operating room scheduling and PACU throughput.

Jeon et al. (63) examined outpatients with mental disabilities undergoing dental treatment, comparing propofol-based TIVA versus propofol converted to remimazolam with flumazenil reversal administered near the end of surgery (63). This hybrid approach demonstrated reduced PACU duration while maintaining procedural sedation quality, representing a pragmatic strategy that leverages the pharmacological advantages of both agents.

Sato et al. (64) compared remimazolam-flumazenil versus propofol for awake craniotomy using an asleep-awake-asleep technique (64). Remimazolam demonstrated faster arousal times (54.48 ± 3.45 vs. 99.22 ± 11.78 s to first eye opening, *p* = 0.0014) and improved intraoperative task performance during the awake phase. This application highlights remimazolam’s potential in neurosurgical settings where rapid, reliable arousal is clinically essential.

Koch et al. (65) described a promising “switch-and-antagonize” approach in a five-patient case series: combined propofol-sevoflurane-remimazolam anesthesia was transitioned to remimazolam (0.9–1.0 mg/kg/h) as the sole hypnotic near surgery end, followed by flumazenil 0.5 mg antagonism. All patients achieved rapid, predictable emergence free from excitation or hemodynamic and respiratory disturbances, with minimal postoperative opioid requirements and no anti-emetic use, suggesting the feasibility of integrating flumazenil reversal into multi-agent anesthetic protocols (65).

While the clinical evidence demonstrates favorable efficacy and safety profiles for remimazolam-flumazenil, several safety considerations warrant specific attention, particularly regarding historical concerns about seizure risk, formulation-specific issues, and the management of special patient populations.

## Safety considerations

### Seizure risk: reconciling pharmacovigilance and clinical evidence

Historical concerns regarding flumazenil-associated seizures derive primarily from observations in patients with chronic benzodiazepine dependence or mixed overdose presentations (66, 67). The mechanistic basis involves abrupt reversal of chronic GABAergic inhibition, precipitating withdrawal-like neuronal hyperexcitability in dependent individuals.

#### Pharmacovigilance data

An and Jiang (68) analyzed the FDA Adverse Event Reporting System (FAERS) database spanning 2004–2023 and identified a substantially elevated signal for withdrawal seizures associated with flumazenil, with a Reporting Odds Ratio (ROR) of 1737.25 (95% CI 1532.08–1969.71) (68). This extraordinarily high signal requires careful interpretation within the context of FAERS limitations and reporting biases.

#### Protopathic bias interpretation

The elevated FAERS signal likely reflects protopathic bias (reverse causation) and confounding by indication. In emergency and overdose settings, flumazenil is administered to patients presenting with altered mental status from mixed drug ingestions; these patients independently carry elevated seizure risk due to co-ingestants [cyclic antidepressants were present in 42% of reported seizure cases in Spivey’s foundational analysis (59)], benzodiazepine dependence, or pre-existing seizure disorders. The temporal association between flumazenil administration and seizure occurrence does not establish causality, as FAERS reports capture co-occurrence without accounting for pre-existing risk factors or the clinical indication for flumazenil use.

#### Surgical database evidence

Komatsu et al. (69) conducted a large-scale analysis using the Japanese Diagnosis Procedure Combination database, comparing seizure incidence between remimazolam-flumazenil and propofol TIVA in 12,033 and 432,275 patients, respectively (69). After propensity score matching, no significant difference in perioperative seizure incidence was observed (adjusted OR 1.08; 95% CI 0.49–2.37). This large real-world dataset provides reassurance that flumazenil reversal following remimazolam does not substantially increase seizure risk in appropriately screened elective surgical patients.

#### Neurophysiological considerations

While epidemiological confounding explains much of the FAERS signal, any rapid reversal of GABAA receptor agonism induces acute neuronal excitability. Abrupt withdrawal of GABAergic tone can theoretically lower seizure threshold even in neurologically healthy brains, particularly in the context of pre-existing factors that compromise inhibitory neurotransmission. Concurrent administration of pro-convulsant medications (tranexamic acid, fluoroquinolones, tramadol) may amplify this risk (70, 71). Preoperative screening for chronic benzodiazepine use remains essential.

#### Formulation-specific considerations

Remimazolam besylate formulations contain Dextran 40 as an excipient, which is contraindicated in patients with known severe dextran hypersensitivity (32, 72). Although no published case has documented Dextran 40 anaphylaxis being confused with sedation-related adverse events during remimazolam use, the clinical presentations can overlap: Lee and Kim (73) reported that hypotension occurred in 81.8% and desaturation in 36.4% of remimazolam-associated anaphylaxis cases, symptoms also recognized as sedation-related adverse events (73). Clinicians should be aware that hemodynamic deterioration unresponsive to flumazenil may indicate a non-benzodiazepine etiology, including excipient-related anaphylaxis, and should prompt standard anaphylaxis management rather than additional flumazenil doses. Patients with documented severe dextran reactions should receive alternative sedatives. Additionally, Ye et al. (74) identified a substantially elevated pharmacovigilance signal for anaphylactic shock (Reporting Odds Ratio 105.97) and anaphylactic reaction (ROR 68.03) associated with remimazolam in the FAERS database, reinforcing the importance of anaphylaxis preparedness and differential diagnosis when flumazenil fails to reverse hemodynamic compromise (74).

The remimazolam formulation contains Dextran 40 as an excipient, a consideration relevant to reversal practice because anaphylactoid reactions occurring during remimazolam infusion may mimic or complicate the clinical picture of sedation-related adverse events, potentially confounding the decision to administer flumazenil. Hypersensitivity reactions to dextran can occur through distinct mechanisms, including IgG-mediated immune complex activation and direct complement pathway stimulation, which are independent of the sedative pharmacology of remimazolam itself. Patients with documented severe dextran reactions should receive alternative sedatives.

## Special populations

### Hepatic impairment

Remimazolam clearance is reduced to approximately 25% of normal in patients with Child-Pugh C cirrhosis, reflecting impaired CES1 activity in advanced liver disease (31). Stöhr et al. (31) characterized pharmacokinetics across hepatic impairment severity, demonstrating progressively prolonged elimination half-life and increased area under the curve with advancing hepatic dysfunction.

Clinical implications include the need for reduced initial dosing, extended intervals between supplemental doses, and prolonged post-procedural monitoring. The response to flumazenil reversal in hepatically impaired patients has not been systematically evaluated, though theoretical concerns exist regarding both delayed remimazolam clearance and potentially altered flumazenil disposition in patients with significant hepatic dysfunction.

### Elderly patients

Elderly patients exhibit both pharmacokinetic and pharmacodynamic alterations affecting remimazolam response. Reduced clearance, increased volume of distribution (related to altered body composition), and enhanced receptor sensitivity collectively result in increased drug effect for a given dose (75, 76). Population pharmacokinetic models have identified age as a significant covariate affecting remimazolam disposition.

Toyota et al. (77) specifically evaluated remimazolam-based anesthesia with flumazenil reversal in elderly patients, demonstrating faster emergence (4 vs. 8 min) compared with propofol but did not systematically evaluate re-sedation rates (77). The median effective dose for induction was substantially lower in patients over 75 years (approximately 0.06 mg/kg) compared with younger adults (approximately 0.09 mg/kg) (75).

Extended post-reversal monitoring is particularly important in elderly patients given increased pharmacodynamic sensitivity and the potential for delayed emergence. The meta-analysis by Ahmer et al. (52) confirmed remimazolam’s favorable safety profile in elderly patients, supporting its consideration in this vulnerable population (52). Liu et al. (56) conducted a meta-analysis of 8 RCTs (571 elderly participants) demonstrating reduced hypotension (RR 0.51) and bradycardia (RR 0.56) with remimazolam versus propofol, while explicitly identifying the lack of elderly-specific flumazenil reversal data as a critical evidence gap requiring investigation (56).

### Neonates and infants

#### Evidence status

Clinical pharmacokinetic data for remimazolam in infants < 1 year do not exist. Recently, Colin et al. (78) published the first population pharmacokinetic study in pediatric patients, but this study included children and adolescents aged 6–18 years, excluding neonates and young infants (78). In addition, Gao et al. (79) characterized remimazolam pharmacokinetics in 24 children aged 2–6 years, reporting weight-normalized clearance of 15.9 mL/kg/min and a context-sensitive half-time of approximately 17 min, suggesting pediatric pharmacokinetic properties broadly similar to adults when normalized to body weight (79). Emerging evidence now extends to flumazenil reversal in pediatric patients: Lee et al. (80) reported the first published cases of two adolescents (ages 12–13) undergoing spinal fusion in whom incremental flumazenil (0.1 mg then 0.2 mg) achieved command-following within 1–3 min, recommending pediatric dosing of 0.01–0.02 mg/kg increments with a maximum single bolus of 0.1–0.2 mg (80). Zhang et al. (81) conducted a systematic review of 23 studies (2,847 pediatric patients) reporting a 15% re-sedation rate post-flumazenil and identified the CES1 G143E polymorphism as a pharmacogenomic basis for prolonged sedation, with carriers exhibiting greater than 90% reduction in clearance (81).

#### Developmental pharmacology concerns

CES1 enzyme expression demonstrates significant developmental regulation, with activity substantially reduced in neonates and approaching adult levels by approximately 12 months of age (82, 83). This enzymatic immaturity would impair parent drug metabolism, potentially resulting in prolonged sedation duration and unpredictable pharmacokinetics.

#### Metabolite accumulation and mass action concerns

The metabolite CNS 7054 is eliminated renally. Neonatal glomerular filtration rate (20–40 mL/min/1.73 m^2^ at birth) is markedly reduced compared with adults (84). As noted above, CNS 7054 has approximately 300-fold lower receptor affinity than remimazolam (3). However, pharmacological principles suggest that mass action can theoretically overcome reduced affinity: if renal immaturity causes metabolite concentrations to accumulate substantially above adult levels, CNS 7054 could hypothetically achieve clinically relevant receptor occupancy. This remains a hypothesis-generating concern without supporting clinical data, and the actual magnitude of neonatal metabolite accumulation has not been characterized.

Assuming the metabolite retains agonist efficacy at the GABAA receptor (rather than functioning as a partial agonist or antagonist), this creates theoretical risk of “metabolite-induced sedation” that is mechanistically distinct from parent drug effects and would not respond to flumazenil antagonism. The intrinsic efficacy of CNS 7054 has not been separately characterized in published literature.

#### Recommendation

Given the complete absence of pharmacokinetic data, risks of both impaired parent drug metabolism and metabolite accumulation, use of remimazolam in infants < 1 year is not recommended outside research settings with appropriate ethics approval and intensive monitoring capabilities.

## Clinical practice considerations

Note: The following represents evidence synthesis and theoretical reasoning derived from pharmacokinetic-pharmacodynamic principles, not validated clinical guidelines. Formal guidelines require systematic review methodology and expert consensus processes.

### Re-sedation risk assessment and management

Based on pharmacokinetic modeling and clinical evidence synthesis, factors associated with increased re-sedation vulnerability may include: prolonged infusion duration (> 60 min), high cumulative remimazolam dose, advanced age (> 75 years), hepatic impairment, reduced CES1 activity (whether genetic or drug-induced), and large flumazenil bolus doses (> 0.5 mg).

Conversely, factors associated with lower re-sedation risk may include: short procedural sedation (< 30 min), low cumulative remimazolam dose, younger age with normal hepatic function, and incremental flumazenil titration rather than large boluses.

Management of suspected re-sedation should follow a systematic approach: (1) Assess airway patency and ventilatory adequacy, (2) Consider whether observed sedation may represent unmasking of opioid effect rather than benzodiazepine recurrence, (3) Administer supplemental flumazenil 0.2 mg if benzodiazepine re-sedation is suspected, (4) Consider naloxone if opioid contribution is likely, (5) Extend monitoring duration proportional to risk factor burden.

### Flumazenil administration strategies

Based on pharmacokinetic-pharmacodynamic modeling (42) and regulatory labeling recommendations, incremental flumazenil dosing (0.2 mg boluses at 60-s intervals) may produce smoother antagonist kinetics compared with large single boluses, potentially reducing the magnitude of post-reversal pharmacokinetic mismatch (42, 85). The titration endpoint should be adequate arousal (response to verbal commands, purposeful movement) rather than complete reversal to baseline alertness.

For elderly or hepatically impaired patients, consider extended intervals between flumazenil doses (2–3 min) to allow assessment of peak effect before additional dosing. Avoid flumazenil boluses exceeding 0.5 mg to minimize the pharmacokinetic mismatch phenomenon. Total flumazenil doses rarely need to exceed 1 mg for reversal of procedural sedation-level remimazolam exposure. Notably, Kim et al. (86) demonstrated through a biased-coin up-and-down dose-finding study that the ED90 of flumazenil for selectively reversing respiratory depression without reversing consciousness was approximately 76.7 μg (95% CI: 68.07–102.62 μg), suggesting that ultra-low-dose flumazenil may rescue respiratory depression during ongoing sedation without terminating the procedure (86).

These recommendations derive from theoretical reasoning and clinical experience rather than prospective randomized trials comparing specific flumazenil dosing protocols. The optimal flumazenil dosing strategy for remimazolam reversal represents an important evidence gap requiring dedicated investigation. Suzuki et al. (87) demonstrated in a double-blind RCT that while flumazenil accelerated consciousness recovery after remimazolam, it did not improve psychomotor or equilibrium function; time until safe discharge remained 120 min regardless of flumazenil use (87). This finding underscores that reversal of sedation level does not equate to functional recovery, and post-reversal monitoring intervals should be determined by objective functional assessments rather than consciousness level alone.

## Evidence gaps and future research directions

This review identifies several critical gaps requiring further investigation:

(1) Prospective validation of pharmacokinetic-pharmacodynamic models against clinical re-sedation outcomes. Current theoretical frameworks derive primarily from simulation studies and retrospective analyses; prospective studies correlating predicted vulnerability windows with objectively measured re-sedation events are needed.
(2) Standardized re-sedation definitions established through Delphi consensus methodology. The current heterogeneity in outcome definitions precludes meaningful synthesis of re-sedation incidence across studies.
(3) Pediatric pharmacokinetic studies, particularly in neonates and infants where developmental CES1 immaturity creates unique pharmacological considerations.
(4) Characterization of CNS 7054 intrinsic efficacy at GABAA receptors and investigation of metabolite accumulation kinetics in renal impairment.
(5) Comparative flumazenil dosing protocols examining incremental versus bolus administration strategies for optimal balance between reversal speed and re-sedation risk.
(6) Clinical translation of CES1 pharmacogenomic findings to determine whether genetic testing provides actionable information for remimazolam dosing or monitoring.
(7) Long-term outcome studies examining whether the cardiorespiratory safety advantages observed during procedural sedation translate into meaningful downstream clinical benefits relevant to reversal practice (e.g., reduced unplanned admissions attributable to post-reversal monitoring protocols).

## Conclusion

Flumazenil reversal of remimazolam offers potential for active sedation termination that is unavailable with propofol-based protocols. Recent high-quality meta-analyses demonstrate clinically meaningful efficacy with significantly improved cardiorespiratory safety profiles, including significant reductions in respiratory depression (RR 0.41) and treatment-requiring hypotension (RR 0.25) compared with propofol (9), predominantly demonstrated in gastrointestinal endoscopy settings. The generalizability of these findings to other procedural contexts and higher-acuity settings requires further investigation.

Re-sedation rates of 2–22% indicate the continued need for post-reversal monitoring, with risk magnitude influenced by cumulative drug exposure, infusion duration, patient factors (age, hepatic function), and potentially CES1 pharmacogenomics. Clinicians should recognize that apparent post-reversal sedation may represent unmasking of residual opioid effects rather than true benzodiazepine recurrence, informing appropriate management with naloxone when indicated.

The re-sedation mechanism involves complex pharmacokinetic-pharmacodynamic interactions including antagonist redistribution, effect-site equilibration kinetics, and receptor occupancy dynamics. Current theoretical frameworks derive from simulation studies and await prospective clinical validation.

The apparent discrepancy between elevated FAERS pharmacovigilance signals and surgical database evidence for seizure risk supports the hypothesis of confounding by indication in emergency settings. In appropriately screened elective surgical patients without benzodiazepine dependence, flumazenil reversal following remimazolam does not appear to substantially increase seizure risk.

Taken together, the available evidence supports the conclusion that flumazenil reversal meaningfully modifies the risk-benefit profile of remimazolam sedation: it confers the unique advantage of active, on-demand sedation termination while introducing manageable risks (re-sedation, theoretical seizure concern in dependent patients) that can be mitigated through incremental dosing, appropriate patient screening, and structured post-reversal monitoring. This narrative review identifies evidence patterns and critical gaps; formal clinical guidelines require systematic review methodology and expert consensus processes. The remimazolam-flumazenil combination represents a valuable addition to the anesthetic armamentarium when used with appropriate understanding of its pharmacological characteristics and limitations.
